# Combination of anti-CGRP/CGRP-R mAbs with onabotulinumtoxin A as a novel therapeutic approach for refractory chronic migraine: a retrospective study of real-world clinical evidence and a protocol for a double-blind, randomized clinical trial to establish the efficacy and safety

**DOI:** 10.3389/fphar.2023.1296577

**Published:** 2023-12-13

**Authors:** M. T. Corasaniti, G. W. Lawrence, G. Bagetta, R. Iannacchero, A. Tarsitano, A. Monteleone, M. Pagliaro, P. Tonin, G. Sandrini, P. Nicotera, D. Scuteri

**Affiliations:** ^1^ Department of Health Sciences, University “Magna Graecia” of Catanzaro, Catanzaro, Italy; ^2^ Department of Biotechnology, Dublin City University, Dublin, Ireland; ^3^ Pharmacotechnology Documentation and Transfer Unit, Preclinical and Translational Pharmacology, Department of Pharmacy, Health and Nutritional Sciences, University of Calabria, Rende, Italy; ^4^ Department of Neurology, Headache Center, Regional Hospital “Pugliese-Ciaccio”, Catanzaro, Italy; ^5^ Pain Therapy Center, Provincial Health Authority (ASP), Cosenza, Italy; ^6^ Regional Center for Serious Brain Injuries, S. Anna Institute, Crotone, Italy; ^7^ Department of Brain and Behavioral Sciences, IRCCS C. Mondino Foundation Neurologic Institute, University of Pavia, Pavia, Italy; ^8^ German Center for Neurodegenerative Diseases (DZNE), Bonn, Germany

**Keywords:** onabotulinumtoxin A, chronic migraine, anti-CGRP monoclonal antibodies, anti-CGRP-R monoclonal antibodies, erenumab, eptinezumab

## Abstract

Chronic migraine is a disabling neurovascular disorder that ranks amongst the top causes of years lived with disability worldwide. The duration and the frequency of migraine affect cognitive and affective domains, inducing worsening of memory, executive functions, orientation and causing anxiety. Population-based studies report a worrying level of resistance to treatments. Therefore, this study aims: 1) to assess efficacy of monoclonal antibodies (mAbs) directed towards the calcitonin gene-related peptide (CGRP) or its receptor (CGRP-R) for chronic migraine resistant to current preventatives; 2) to design a clinical trial protocol to evaluate the efficacy and safety of combination therapy utilizing anti-CGRP/CGRP-R together with onabotulinumtoxin A in patients suffering from resistant chronic migraine; 3) to provide a molecular rationale for combination therapy. A controlled trial is warranted as pooled analysis of real-world data from our group highlighted that combined treatment provides ≥50% reduction vs. baseline (onabotulinumtoxin A) of monthly headache days (MHDs) in up to 58.8% of patients, but there has been only sparse application of this combined therapy to date. The mAbs chosen are: erenumab, because its combination effect with onabotulinumtoxin A improved symptoms in 65% of patients; eptinezumab, due to its faster action. The results highlight that early diagnosis of migraine improves therapeutic outcomes with mAbs alone, confirming their effectiveness and the need for an adequately powered clinical trial evaluating the safety and potential superior effectiveness of eptinezumab/erenumab and onabotulinumtoxin A together.

## 1 Introduction

### 1.1 Migraine and refractoriness

Migraine is a disabling, primary headache endowed with a serious social impact, ranking as one of the top causes of years lived with disability worldwide, particularly in people under fifty (Steiner et al., 2018), is 3-to-4 times more frequent in females than in males (Rossi et al., 2022). Chronic migraine is a disease characterized by episodic manifestations (Haut et al., 2006), which the International Classification of Headache Disorders (ICHD, third revision) beta diagnostic criteria defines as at least 15 headache days per month, of which 8 days present the features of migraine, for 3 months consecutively (Headache Classification Committee of the International Headache Society, 2013). The social burden of chronic migraine is increased by its remarkable undertreatment and a high prevalence of resistance to current treatments such as the widely used triptans that stimulate serotonin receptors and the anti-epileptic topiramate which suppresses electrical overactivity in the central nervous system (Schulman et al., 2008). The mechanisms causing resistance to treatments have not yet been elucidated, but a role for genetic polymorphisms has been highlighted (Scuteri et al., 2021b). The duration and the frequency of migraine correlates with harm in both the cognitive and affective domains, damage to memory, executive functions and of orientation, as assessed through the Rey–Osterrieth complex figure test (ROCF) and the Montreal Cognitive Assessment (MoCA), and causing anxiety (Huang et al., 2017). Reduction of monthly headache days (MHDs) and monthly migraine days (MMDs) is a main goal of treatments, but the difficulty in treating chronic migraine in a large proportion of patients has prompted the development of novel therapies.

### 1.2 Game-changing novel therapies

The discovery of the involvement of the calcitonin gene-related peptide (CGRP) in the pathogenesis of migraine (Scuteri et al., 2019a) fostered the advance of novel specific small molecules (Scuteri et al., 2022b; Scuteri et al., 2022c) and biotechnological drugs (Scuteri et al., 2019b; Scuteri and Bagetta, 2021) targeting this peptide (Scuteri et al., 2020). Onabotulinumtoxin A has been approved since 2010 for the treatment of chronic migraine relying on data from the Phase III Research Evaluating Migraine Prophylaxis Therapy (PREEMPT) clinical program (Diener et al., 2010; Aurora et al., 2011; Aurora et al., 2014). This drug inhibits the exocytosis of CGRP from primary sensory neurons, as well as the release of several other neurotransmitters (Dolly, 2003; Aoki, 2005) by cleavage of the 25 kDa synaptosomal-associated protein (SNAP-25) (Welch et al., 2000). It is effective, well-tolerated (Herd et al., 2018) and despite a lack of homogeneous and long-term data, the available results indicate that it is safer than one of the most commonly used preventative drugs, topiramate. In fact, topiramate was reported to be associated to the highest rate of drop-out in comparison with onabotulinumtoxin A and the most novel antibodies (Frank et al., 2021) and with teratogenicity (Rothrock et al., 2019). Hence, more trials are needed to assess the effect of topiramate in chronic migraine with medication overuse (Giri et al., 2023). Side effects of onabotulinumtoxinA are rare, mild, self-limiting and usually resolve within a short time when used as directed by the label, but the potential for unwanted neuromuscular and/or autonomic side-effects precludes increasing the doses used above those specified in the label instructions. The most novel biotechnological drugs (authorized between 2018 and 2020) are the specific monoclonal antibodies (mAbs) directed towards CGRP (known as eptinezumab, galcanezumab and fremanezumab) or its receptor complex (erenumab). However, 38% of patients that failed all the available preventative drugs also did not respond to erenumab after 6 months of treatment (Lambru et al., 2020; Sacco et al., 2021). Such a large refractory group prompted the investigation of a treatment regime combining onabotulinumtoxin A and anti-CGRP mAbs.

### 1.3 Combined therapy with onabotulinumtoxin A and mAbs targeting the CGRP machinery

This afforded a pooled ≥50% reduction of MHDs with respect to baseline (onabotulinumtoxin A injections (Toni et al., 2021) of ≥ 2 consecutive cycles of (Blumenfeld et al., 2021; Mechtler et al., 2022)) in up to 58.8% of patients, with a decline of 35.5% after the 6th month (Scuteri et al., 2022d). Moreover, the combined therapy was more effective than erenumab, administered alone or with other preventative drugs, with the efficacy being prolonged by an average of 2 weeks, a fundamental improvement for refractory patients (Ailani and Blumenfeld, 2022). Nevertheless, head-to-head comparisons are still needed (Lu et al., 2021). The recent American Headache Society (AHS) consensus statement of 2021 (Ailani et al., 2021) reported the possible efficacy of the combination of CGRP-targeted mAbs and onabotulinumtoxin A for patients suffering from continued migraine and disability on a single preventive treatment, occurring when experiencing ≥4 MMDs with at least moderate disability, assessed as Migraine Disability Assessment ≥11, Headache Impact Test >50, or ≥8 MMDs (Ailani and Blumenfeld, 2022). Therefore, the purposes of the present study are: 1) to assess the resistance of chronic migraine to current preventative drugs and the consequent use and efficacy of the anti-CGRP/CGRP-R mAbs in a real-world setting; 2) to design a clinical trial protocol to evaluate the effectiveness and tolerability of combination therapy for resistant chronic migraine utilizing anti-CGRP/CGRP-R together with onabotulinumtoxin A; 3) to provide a molecular rationale for such combination therapy.

## 2 Methods

### 2.1 Objectives of the retrospective phase

This retrospective study was conducted in collaboration with the Pain Therapy Center of the Provincial Health Authority of Cosenza (Calabria, Italy). Anonymized data were collected concerning the following aspects: demographic characteristics of the patients; diagnosis of chronic migraine according to the ICHD 3 beta; failed preventative treatments; anti-CGRP mAbs administered; baseline MMDs; reduction of MMDs after 1, 3 and 6 months of treatment with mAbs; decrease of pain intensity measured by the numeric rating scale (NRS) after 6 months of treatment. The district consists of 298,000 inhabitants, 213,000 under 60 years of age, i.e., a sample typical for migraine occurrence. The patients enrolled in the study had no concomitant pathologies and were not undergoing concurrent treatments. The need for written informed consent and ethical approval was waived owing to the retrospective use of anonymized data only. The study was conducted in accordance with the Declaration of Helsinki.

### 2.2 Trial design

**FIGURE 1:**
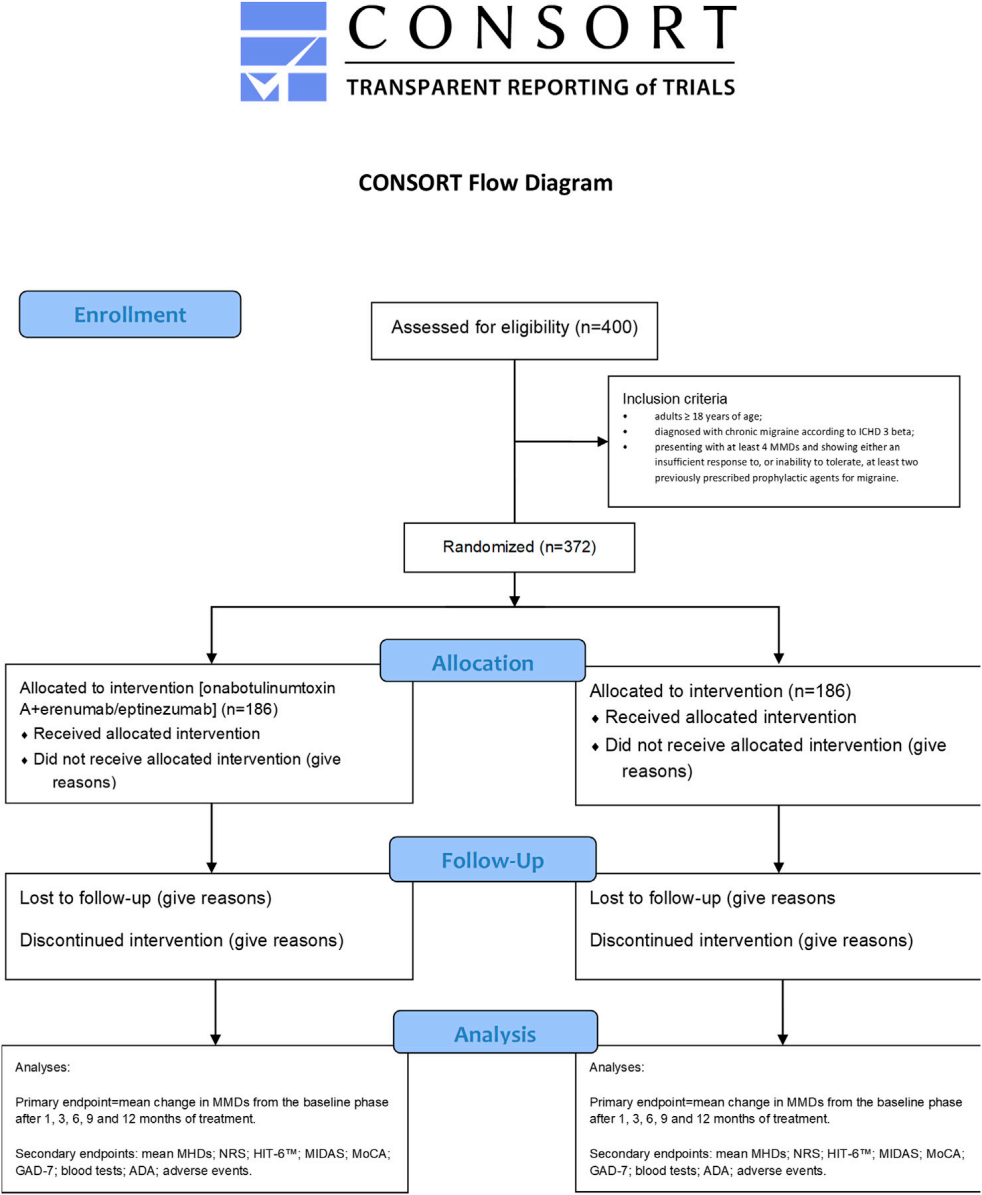
Consolidated Standards of Reporting Trials (CONSORT) flow diagram of the proposed clinical trial.

The trial assessing the efficacy and safety of the combined treatment with onabotulinumtoxin A and either erenumab or eptinezumab will be a double-blind, randomized single-center trial recruiting patients eligible for the intervention, i.e., those suffering from chronic migraine (according to the ICHD 3 beta) that are refractory to the most commonly used preventative treatments (see below for details). The patients allocated to the combination therapy arm of the study will be assigned randomly to erenumab or eptinezumab subgroups, whereas those allotted to the control arm will continue with their usual treatment. The study protocol included implements the Standard Protocol Items: Recommendations for Interventional Trials (SPIRIT) Checklist (Chan et al., 2013) and the Consolidated Standards of Reporting Trials (CONSORT) statement (Moher et al., 2010). It has been submitted for approval to the Calabria Region Ethics Committee and a request has been made for a ClinicalTrials.gov ID. According to the D.lgs 196/2003, the Helsinki agreements and subsequent amendments, the Good Clinical Practice and Guidelines for the treatment of personal data in clinical trials of 24 July 2008, and in accordance with European data protection legislation, each participant or his/her legal representative will be required to sign a consent form as acceptance of all aspects of the study contained in the patient information sheet and as a consequent expression of his/her willingness to participate in the study. The information sheet will be explained to the subjects or legal representatives by the study staff and the same staff will ensure that the consent form is properly signed and dated by all the parties involved before any procedure detailed in the protocol is carried out. Information on opt-out will be provided to the subjects or legal representatives by the study staff. The primary endpoint will be the mean change in MMDs from the baseline phase after 1, 3, 6, 9 and 12 months of treatment. The secondary endpoints are as follows: mean MHDs; numeric rating scale (NRS); six-item Headache Impact Test (HIT-6™); migraine disability assessment (MIDAS); MoCA; GAD-7; blood tests; anti-drug antibodies (ADA); safety end points that, according to usual safety assessment in clinical trials for chronic migraine with anti-CGRP mAbs, include patient incidences or exposure-adjusted patient incidences of adverse events (AEs) and serious AEs, identifying cardiovascular and cerebrovascular risk factors, such as diabetes, at baseline (Ashina et al., 2022). A complete CONSORT flow diagram for the study is reported in Figure 1.

### 2.3 Inclusion criteria

Patients eligible for inclusion in the present clinical trial are:

• adults ≥ 18 years of age;
• diagnosed with chronic migraine according to ICHD 3 beta;
• presenting with at least 4 MMDs and showing either an insufficient response to, or inability to tolerate, at least two previously prescribed prophylactic agents for migraine.

### 2.4 Data analysis

Data for the retrospective observational study were extracted from anonymized migraine diaries of the patients and case report forms, then collated using Microsoft Office Excel 2010 (Microsoft, Milan, Italy). Statistical analyses on data expressed as percentage of reduction relative to baseline were performed using GraphPad Prism^®^ 6.0 (GraphPad software Incorporated, San Diego, CA, United States). The results were statistically evaluated for differences using χ2 test for categorical variables considering *p* < 0.05 significant. The prespecified statistical analysis plan (SAP) for the combination therapy clinical trial consists of an assessment of differences for both the primary and the secondary endpoint measures, according to the calculation of the least-squares mean at each timepoint, evaluated through a linear mixed effects model including all the patient-level variables (Tepper et al., 2017).

## 3 Results

### 3.1 Effectiveness of anti-CGRP/R mAbs in a small sample in the real-world setting

The present retrospective, observational study identified *n* = 10 patients (9 females and 1 male) aged 38–66 years at the time of data collection (2022) that met the inclusion criteria after referral to the Pain Therapy Center of the Provincial Health Authority of Cosenza (Calabria, Italy) in the preceding decade (2010–2022). The gender distribution in this cohort is compatible with literature reporting that migraine is 3-to-4 times more frequent in females than in males (Rossi et al., 2022). The baseline MHDs and MMDs ranged from 15 to 25 and all the patients suffered from chronic migraine without aura (Table 1). Baseline migraine pain intensity was moderate to severe with NRS values from 7 to 10. The patients had all used two or three preventative drugs for an average of 7 years, and failed in the prophylaxis of chronic migraine (patient 017 even showed a worsening of MMDs), before then switching to anti-CGRP mAbs. Although patients 015 and 024 displayed a noteworthy reduction in MMDs with preventative drugs, they elected mAb therapy because their post-treatment MMDs remained high. The drugs used were a selection from the following: topiramate, amitriptyline, flunarizine, verapamil, propranolol, levosulpiride and gabapentin (Table 1). The baseline features of the patients recruited are illustrated in Table 1.

**TABLE 1:** Baseline characteristics of patients included in the study. The cohort were of predominantly females, with a late age of first chronic migraine observation that failed to achieve an adequate reduction of monthly migraine days (MMDs) with current preventative treatments.

Patient code	Age at the time of the study	Sex	Baseline MMDs	Preventative treatments	Post-treatment MMDs	Treatment period
011	43	F	20	Topiramate	20	2013–2021
				Flunarizine		
012	40	F	15	Topiramate	15	2011–2022
				Amitriptyline		
				Verapamil		
014	40	F	22	Propranolol	20	2017–2022
				Topiramate		
				Amitriptyline		
015	46	F	25	Flunarizine Topiramate	17	2014–2022
				Amitriptyline		
016	45	F	18	Gabapentin	18	2017–2022
				Amitriptyline		
				Topiramate		
017	66	F	15	Verapamil	20	2015–2022
				Amitriptyline		
				Levosulpiride		
018	38	F	20	Amitriptyline	20	2014–2021
				Topiramate		
				Flunarizine		
022	58	F	18	Topiramate	18	2010–2020
				Amitriptyline		
023	46	M	25	Propranolol	22	2016–2021
				Gabapentin		
				Amitriptyline		
024	47	F	20	Propranolol	15	2019–2022
				Gabapentin		
				Topiramate		

Six out of the 10 patients eligible for anti-CGRP/R mAbs treatment received monthly administrations of erenumab with titration from 70 mg to 140 mg in two cases, while two patients in treatment with 225 mg of fremanezumab monthly and the other 2 patients with monthly galcanezumab titrated from 240 mg loading dose to 120 mg. Data are reported in Table 2. Nine of the ten patients reported a reduction of MMDs after 1 month of treatment and all recorded a reduction upon assessment after 3 and 6 months of treatment, confirming efficacy. Moreover, 8 of the 10 patients scored a reduced pain intensity at 6 months. Interestingly, older patients (017 and 022) showed more resistance to anti-CGRP mAbs in terms of a less pronounced reduction of MMDs with respect to the whole sample, although the possible age-dependency for treatment outcomes will require further investigation in a larger sample because 2 of the younger patients (012 and 023) also exhibited similarly small reductions in MMDs. Eptinezumab and onabotulinumtoxin A were not used in this sample taken from real-world clinical practice. A mean 42% reduction of MMDs was observed with erenumab producing a 41.83% decline, fremanezumab 41.5% and galcanezumab 42.5%. Data are summarized in Table 2.

**TABLE 2:** Reduction of monthly migraine days (MMDs) and of migraine pain intensity (NRS) after 1, 3 and 6 months of treatment with mAbs targeting either the CGRP (fremanezumab or galcanezumab) or the receptor (erenumab).

Patient code	Antibody	Dose	Frequency of administration	Baseline monthly migraine days (MMDs)	Reduction of MMDs after 1 month	Reduction of MMDs after 3 months	Reduction of MMDs after 6 months	Percentage reduction of MMDs (%)	Baseline pain intensity measured through numeric rating scale (NRS)	Pain intensity NRS after 6 months
011	Fremanezumab	225 mg	Monthly	20	(-5)	(-14)	(-15)	75	8	5
012	Erenumab	70 mg	Monthly	15	(-3)	(-3)	Loss to follow-up	20	10	5
		140 mg								
014	Erenumab	70 mg	Monthly	20	(-10)	(-13)	(-13)	65	10	7
015	Erenumab	70 mg	Monthly	17	(-5)	(-7)	(-7)	41	10	7
016	Erenumab	70 mg	Monthly	18	(-3)	(-8)	(-6)	33.3	10	7
		140 mg								
017	Galcanezumab	240 mg (loading dose)	Monthly	20	(-5)	(-5)	(-5)	25	7	7
		120 mg								
018	Galcanezumab	240 mg	Monthly	20	(-10)	(-12)	(-12)	60	10	5
		120 mg								
022	Erenumab	70 mg	Monthly	17	(-2)	(-2)	(-2)	11.7	10	4
023	Fremanezumab	225 mg	Monthly	25	0	(-13)	(-3)	8	10	10
024	Erenumab	70 mg	Monthly	15	(-12)	(-12)	(-12)	80	7	3

### 3.2 Clinical trial protocol for combination therapy of onabotulinumtoxin A and anti-CGRP mAbs

A double-blind, randomized clinical trial will be conducted at the Headache Center of the Regional Hospital “Pugliese-Ciaccio” directed by Dr. Rosario Iannacchero to assess the effectiveness of combination therapy utilizing onabotulinumtoxin A together with an anti-CGRP mAb compared to patients’ continuation with their current treatment with regard to the mAbs to be used in the combination therapy, both erenumab and eptinezumab will be assessed separately in individual subgroups of the study. Applying the structure of the exploratory analysis of patient-reported outcomes (PROs) for superiority of the intervention in the primary endpoint, that consists in the decrease of mean MMDs (from baseline), NCT02066415, a sample size of *n* = 186 patients for the intervention group is required assuming a treatment effect of −1.9 days with a standard deviation of 6.1, providing 85% power using a two-sample t-test with a two-sided significance level of 0.04 (Tepper et al., 2017). Eligible patients will be randomly assigned in ratio 1:1 to the combination therapy or usual treatment group. Patients in the combined therapy group will receive per label administration of onabotulinumtoxin A and one of either erenumab or eptinezumab. In this way it will be possible to cover all the possible mechanisms, responding either to the inhibition of the signaling of the CGRP ligand (eptinezumab) or its receptor (erenumab) and with the fastest onset of action. Patients, administrators, raters and data analysts will be blinded to the assignments to the intervention or usual care groups, although the administration route can differ. In order to obtain long-term efficacy and safety data the trial will last 52 weeks. Demographic characteristics and baseline information of the patients will be collected through a migraine diary to be completed each day with details including incidence of headache; incidence of migraine with or without aura; time of onset of headache; time to resolution of headache; headache intensity assessed as NRS; pain features; migraine symptoms and most bothersome symptom; use of acute drug treatment during aura or headaches. Baseline assessments will be carried out during the first month immediately before allocation to either of the groups detailed in 2.2. The primary endpoint will be the mean change in MMDs from the baseline phase after 1, 3, 6, 9 and 12 months of treatment. MMDs will be identified based on headache duration, symptoms, pain features and use of migraine-specific drugs use. Migraine is defined as follows: headache (with or without aura) lasting for at least 4 h continuously, with two or more pain features (unilateral, throbbing, moderate to severe intensity, or aggravation by exercise or physical activity) or one or more associated non pain features (nausea, vomiting, or photophobia accompanied by phonophobia) (Tepper et al., 2017). Secondary endpoints include: reduction of mean MHDs; decrease of pain severity measured as NRS; improvement in impact of headache and disability evaluated by the HIT-6™ (Ware et al., 2000; Kosinski et al., 2003; Yang et al., 2011) and the MIDAS (Lipton et al., 2001) scores, respectively; decrease of need for rescue medications; assessment of tolerability. Moreover, MoCA and the Generalized Anxiety Disorder Scale 7-item (GAD-7), validated for migraineurs, will be performed to assess the efficacy of treatment on the cognitive and affective domains (Seo and Park, 2015). Blood tests and searches for neutralizing antibodies against the mAbs will be performed at the beginning and at the end of the study. Any adverse events will be recorded on the case report form during the trial. No sponsor will participate in the trial.

## 4 Discussion

### 4.1 Real-world data and delay in diagnosis

According to age-standardized data from the Global Burden of Disease Study 2016, Italy exhibits the highest calculated prevalence of migraine in the world at 20,000 to 21,000 patients per 100,000 inhabitants population (Stovner et al., 2018). Chronic migraine is a debilitating condition consequent to the process of transformation and progression of episodic migraine (Bigal et al., 2006). Therefore, the prevention of migraine chronification and of medication-overuse headache (MOH) is fundamental. For a long time triptans have represented the sole specific treatment for migraine attacks, which offer 2-h sustained freedom from pain to some 18%–50% patients and sustained headache relief at 24 h to some 29%–50% of patients (Cameron et al., 2015). However, the discovery of CGRP as a mediator of chronic migraine pathophysiology (Edvinsson et al., 2018) has caused a revolution in the treatment and prophylaxis of this condition. Data presented herein, gathered from current clinical practice in Calabria in Italy, reports the efficacy of antibody therapies targeting CGRP or its receptor for reducing MHDs, MMDs and NRS pain score. Interestingly, our sample included patients arriving to clinical observation at the age of over 50 years. This highlights a serious delay in diagnosis, in accordance with an underestimation of the problem of migraine (Gupta and Gaurkar, 2022). Notably nine out of the ten patients analyzed showed MMDs decrease after 1 month of treatment and a reduction after 3 and 6 months. Older patients (017 and 022) showed more resistance to anti-CGRP mAbs. Moreover, the eldest patient (at 66 years old) not only proved completely refractory to current prophylactic therapies, the treatments failed to prevent the further deterioration in symptoms, so further investigations into a possible relationship between age and refractory migraine is warranted. Pain processing alters during aging (Hamm and Knisely, 1985; Jourdan et al., 2000; Jourdan et al., 2002) and this could foster new studies investigating the role of natural products with analgesic activity as potentially useful and safe add-on therapies (Scuteri et al., 2021a; Scuteri et al., 2022a). The development of migraine in over 50 year old patients is unusual (Herrero et al., 2013) and, consequently, this population is often neglected from clinical trials for painkillers in general (Bayer and Tadd, 2000). Another issue for refractory migraine treatment, also in the population of over 50-year-old, is MOH induced by the overuse of drugs. By contrast to the experience of the older patients in this study, a recent real-life multicentre analysis of 162 over 65 year-old patients, showing that anti-CGRP mAbs provided a reduction of MMDs ≥ 50% and ≥ 75% to some 57% and 33% patients, respectively (Muñoz-Vendrell et al., 2023). The present clinical practice data analyzed herein highlight a preference to switch patients from non-specific, preventative, small molecules to mAbs rather than onabotulinumtoxin A. Interestingly, no patients were transferred to eptinezumab, in spite of its rapid action. The use of mAbs proved to be effective, but to different extents in individual recipients, a finding that corroborates international data, possibly due to differences in hypothalamic modulation (Torres-Ferrús et al., 2021; Basedau et al., 2022; Iannone et al., 2022). The real-world data on anti-CGRP-mAbs suffer from the following limitations: retrospective collection of data, small sample size, short follow-up periods (Pavelic et al., 2023). This is confirmed by a recent systematic search that included randomized controlled trials reporting the outcomes of change in MHDs, MMDs, ≥50% response rates and change in MOH status (Giri et al., 2023).

### 4.2 Neuropharmacology of resistant, chronic migraine

**FIGURE 2:**
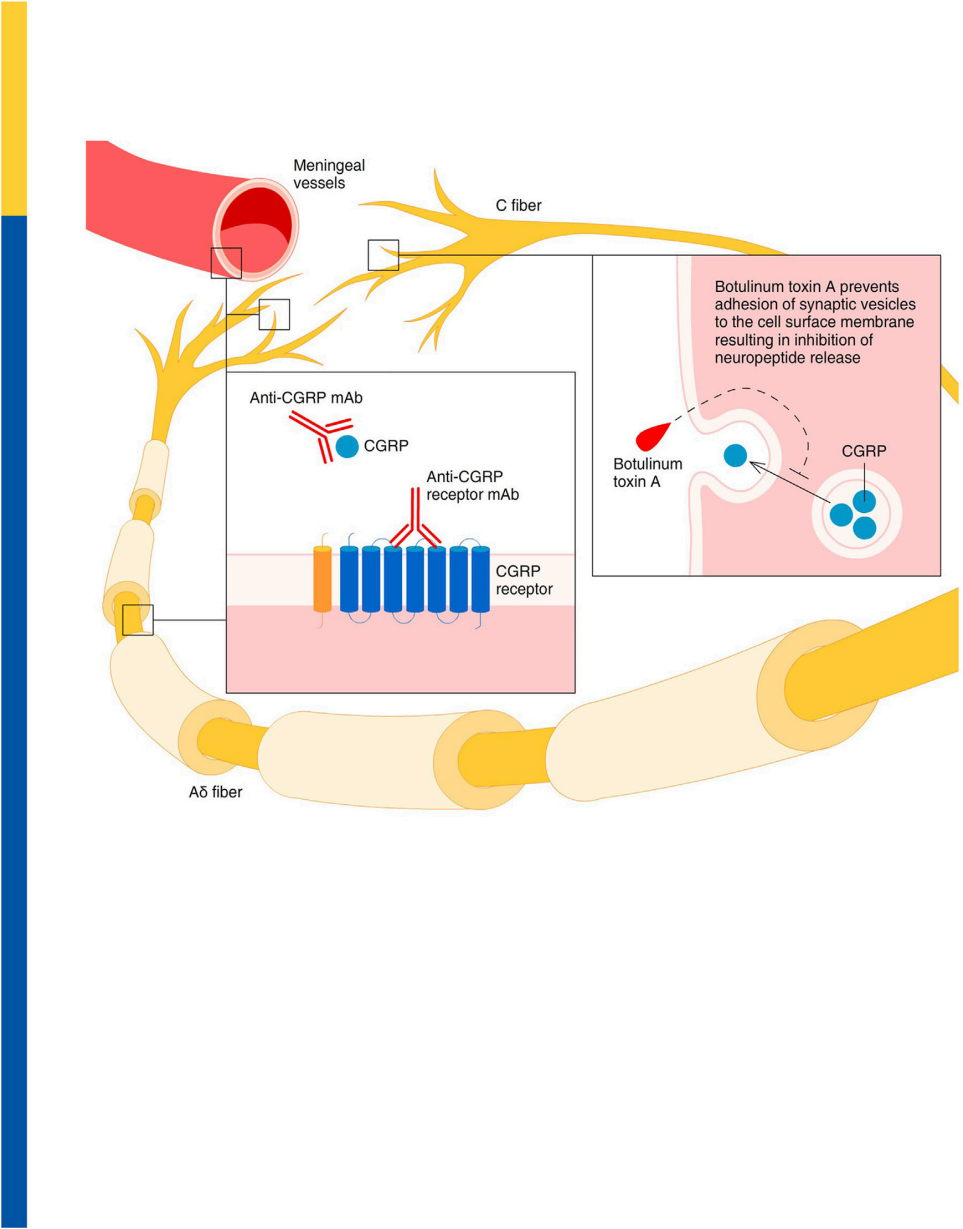
Proposed synergic activity of onabotulinumtoxin A and anti-CGRP mAbs in migraine. Onabotulinumtoxin A inhibits the release of CGRP from thin unmyelinated C fiber meningeal nociceptors in the dura, thus preventing a CGRP-dependent activation of meningeal vessels and thick myelinated Aδ nociceptors. At the same time, anti-CGRP mAbs prevent the interaction between the CGRP and its receptor within the meningeal vessel walls, as well as in the extremities and along the fibers in at the nodes of Ranvier of the Aδ nociceptors. Reproduced with permission from (Pellesi et al., 2020).

As an alternative to mAbs targeting CGRP signalling, onabotulinumtoxin A has been approved for use in chronic migraine since 2010 (Simpson et al., 2016). It proteolyses SNAP-25, one of the proteins required for the membrane fusion reaction that mediates the exocytosis of CGRP, as well as other neuropeptides and various neurotransmitters (Meng et al., 2009; Cernuda-Morollón et al., 2015; Belinskaia et al., 2023). Among these neurotransmitters, it is possible to find acetylcholine, glutamate, pituitary adenylate cyclase activating peptide 38 (PACAP 38) and substance P, inhibition of the trafficking of transient receptor potential cation channel subfamily V member 1 [TRPV1], transient receptor potential cation channel subfamily A member 1 [TRPA1] and purinergic receptor P2X ligand-gated ion channel 3 [P2X3] (Devesa et al., 2014; Meng et al., 2016) is also implicated in the mechanism of action of onabotulinumtoxin A (Burstein et al., 2020). The effect is long-lasting, as supported by the evidence that SNAP-25 is cleaved over 80 days in cultured spinal cord cells (Keller et al., 1999) and a minimum of 3 months is recommended between clinical injections for chronic migraine (BOTOX^®^ label information). Botulinum toxin can be useful in several other types of pain, as including neuropathic. In fact, a double-blind, crossover, pilot trial investigating the effectiveness of botulinum toxin type A for diabetic neuropathy, demonstrated a significant reduction of pain intensity assessed through visual analog scale (VAS) at 1-4-8 and 12 weeks after administration (Yuan et al., 2009). In relation to its mechanism of action, onabotulinumtoxinA reduced interictal plasma levels of CGRP, determined in samples obtained from the right antecubital vein using ELISA, one month after treatment, in chronic migraineurs who are responders to treatment, but not in nonresponders (Cernuda-Morollón et al., 2015). By contrast, the levels of vasoactive intestinal peptide (VIP), investigated in samples obtained from the right antecubital vein by ELISA, were significantly increased in responders (Cernuda-Morollón et al., 2014). The gathered evidence suggests that measurements of the interictal levels of CGRP may be helpful to predict the response to onbaotulinumtoxin A (Cernuda-Morollón et al., 2015), supporting the importance of an additive/synergic mechanism with antibody therapy. The synergic mechanism of the combination in migraine may be related to the inhibition of CGRP release from thin unmyelinated C fiber dural nociceptors produced by onabotulinumtoxin A, while anti-CGRP mAbs prevent the binding of the ligand to its receptor (Figure 2; reproduced with permission from (Pellesi et al., 2020)). This is corroborated by the finding that fremanezumab can prevent the activation of Aδ- but not C-fibers, whilst onabotulinumtoxin A might selectivley interact with C- but not Aδ-fibers, in agreement with distribution of CGRP in C-fibers and CGRP receptors in Aδ-fibers (Strassman et al., 2004; Eftekhari et al., 2013; Pellesi et al., 2020). It has also been proposed that onabotulinumtoxin A reverses mechanical hypersensitivity of sensitized C-units by interference with the expression of high-threshold mechanosensitive ion channels on the surface of nerve cells (Burstein et al., 2014). Thus, onabotulinumtoxin A can be exploited as a multipurpose drug offering long-lasting relief from several forms of pain including migraine (Sandrini et al., 2017; De Icco et al., 2019) and neuropathic pain in experimental conditions (Wang et al., 2017). Accordingly, clinical use of onabotulinumtoxin A has been shown to decrease the need for rescue medications (Sandrini et al., 2011). Chronic migraine is a form of complex, neurological disorder characterized by sensory, cognitive and affective comorbidities, likely due to network disruption (DeSouza et al., 2020), that needs to be prevented to avoid patients’ disability. So to prevent patients’ disability, it is fundamental to provide relief from all these associated modalities. Indeed, a prospective cross-sectional study on 165 patients highlighted that some 89.7% of them experienced cognitive symptoms with consequent dysfunction involved in the attack-related disability (Gil-Gouveia et al., 2016), thus representing a pathological outcome of the utmost importance. This can be, at least in part, due to the perturbations of brain areas important for cognition, i.e., amygdala, hypothalamus, periaqueductal gray (PAG), ventral pontine tegmentum, ventral and dorsal medulla and also spinal cord (Kozlowska et al., 2015; Gil-Gouveia and Martins, 2019). Executive functions, working memory, visual-spatial processing and attention are among the most affected cognitive skills (Gil-Gouveia and Martins, 2019). Cognitive symptoms rank second in the symptoms related to the attack of migraine (Vuralli et al., 2018; Gil-Gouveia and Martins, 2019) and these are accompanied by low-rate depression, anxiety and apathy (Santangelo et al., 2016), supporting the need for long-term assessment of cognitive impairment in chronic migraineurs. CGRP is thought to be a trigger factor for migraine because injections of levels of this neuropeptide can induce migraine-like headache symptoms. Also, in some migraineurs, its levels are elevated (compared to healthy controls) in the inter-ictal phase and have been seen to increase even further during the pre-ictal to headache onset phase (Kamm, 2022, Front. Neurol.). Anti-CGRP mAb therapies, or botulinum toxin injections, are thought to reduce migraine incidence and severity by suppressing interictal and ictal levels of this neuropeptide, thereby, halting migraine progression and alleviating the development of cognitive and affective comorbidity. Hence, the inclusion in the clinical study herein of the MoCA and GAD-7 analyses to investigate the impact of combination therapy on these facets.

### 4.3 Rational basis for co-administration of onabotulinumtoxin A and mAbs directed towards the CGRP ligand or receptor

Notably, retrospective studies indicate that co-administering onabotulinumtoxin A with anti-CGRP/R mAbs can more successfully reduce MMDs and MHDs and prolong the suppression of migraine impact with induced disability than either individual intervention alone. In fact, the combination therapy was associated with statistically significant reductions of 8.1 MHDs (*p* < 0.001) and of 7.4 MMDs [30% (*p* < 0.001)] at 90 days (Armanious et al., 2021). In this regard, an interesting study by Blumenfeld and coworkers of 2021 (Blumenfeld et al., 2021) reported a wide primary analysis cohort (n = 257) and sensitivity analysis cohort (n = 172), including only patients suffering from moderate disability defined by MIDAS score>11 or HIT-6^TM^ score >50. This study demonstrated that, after 6–12 months of combined therapy, one-third (31.5%–36.7%) of patients presented a reduction of MHDs ≥50% and a reduction in migraine-related disability ≥30%. Furthermore, the mean MIDAS score for 27.1%–29.6% of the cohort was significantly reduced from baseline by between 6.1 and 11.1 points. Another retrospective study likewise highlighted the effectiveness of onabotulinumtoxin A as an add-on therapy to mAbs in patients suffering from refractory chronic migraine, who failed two oral migraine preventative drugs, three onabotulinumtoxin A cycles and three sessions with either fremanezumab or erenumab delivered sequentially as monotherapies (Argyriou et al., 2022). Furthermore, addition of an anti-CGRP mAb to the treatment for MOH in chronic migraineurs has been recently suggested to reduce headache frequency and symptomatic medication use (Krymchantowski et al., 2023). Although patient persistence with onabotulinumtoxinA is better than that seen with anti-CGRP mAbs (Schwedt et al., 2023), the doses of botulinum toxin A that can be delivered are strictly limited to restrict the unwanted spread of the toxin beyond the treatment area and to preclude the development of potentially debilitating motor and autonomic side-effects. Consideration of all these data together prompted the design of a prospective clinical trial to evaluate the effectiveness and tolerability of combination treatment using mAbs together with onabotulinumtoxin A (Guerzoni et al., 2022). Therefore, here we propose a study protocol for a 52-week, randomized, adequately powered, clinical trial to provide long-term evidence for effectiveness and tolerability of the combined treatment of onabotulinumtoxin A and anti-CGRP mAbs. The mAbs chosen are erenumab, because its combination beneficial effect was demonstrated in some 65% patients (Boudreau, 2020) and eptinezumab due to its faster action. The main goal of this prospective randomized clinical trial is to fill the gap between clinical practice and research due to the still unmet need for wide prospective clinical trials assessing long-term follow-up of combined therapies and to offer a new therapeutic approach for refractory patients. This trial falls in the novel field considering chronic migraine management of patients with frequent and disabling attacks as a multimodal strategy including the control of the following milestones: 1) comorbidities; 2) modifiable risk factors involved in the process of progression as the overuse of medications and of caffeine; 3) secondary headaches; 4) tailored acute and preventative therapies with the aim of reducing pain, allodynia, cognitive and affective impairment and consequent disability (Blumenfeld et al., 2023). Moreover, statistical modeling deserves further investigation since it can represent an important aid in the prediction of synergistic or additive effects of treatments combinations also in acute management of the attacks (Blumenfeld et al., 2012).

## Data Availability

The original contributions presented in the study are included in the article/Supplementary material, further inquiries can be directed to the corresponding author.
